# Quality assurance for a six degrees‐of‐freedom table using a 3D printed phantom

**DOI:** 10.1002/acm2.12227

**Published:** 2017-11-21

**Authors:** Kyle Woods, Ahmet S. Ayan, Jeffrey Woollard, Nilendu Gupta

**Affiliations:** ^1^ Department of Radiation Oncology Ohio State University Columbus OH USA

**Keywords:** 3D printing, 6DoF, quality assurance, six degrees‐of‐freedom

## Abstract

**Purpose:**

To establish a streamlined end‐to‐end test of a 6 degrees‐of‐freedom (6DoF) robotic table using a 3D printed phantom for periodic quality assurance.

**Methods:**

A 3D printed phantom was fabricated with translational and rotational offsets and an imbedded central ball‐bearing (BB). The phantom underwent each step of the radiation therapy process: CT simulation in a straight orientation, plan generation using the treatment planning software, setup to offset marks at the linac, registration and corrected 6DoF table adjustments via hidden target test, delivery of a Winston‐Lutz test to the BB, and verification of table positioning via field and laser lights. The registration values, maximum total displacement of the combined Winston‐Lutz fields, and a pass or fail criterion of the laser and field lights were recorded. The quality assurance process for each of the three linacs were performed for the first 30 days.

**Results:**

Within a 95% confidence interval, the overall uncertainty values for both translation and rotation were below 1.0 mm and 0.5° for each linac respectively. When combining the registration values and other uncertainties for all three linacs, the average deviations were within 2.0 mm and 1.0° of the designed translation and rotation offsets of the 3D print respectively. For all three linacs, the maximum total deviation for the Winston‐Lutz test did not exceed 1.0 mm. Laser and light field verification was within tolerance every day for all three linacs given the latest guidance documentation for table repositioning.

**Conclusion:**

The 3D printer is capable of accurately fabricating a quality assurance phantom for 6DoF positioning verification. The end‐to‐end workflow allows for a more efficient test of the 6DoF mechanics while including other important tests needed for routine quality assurance.

## 1 INTRODUCTION

The robotic patient positioning table is a vital component in external beam radiation therapy treatments. These tables can mechanically drive a patient to a desired treatment position with the aid of external skin marks or fiducials. Furthermore, most modern linear accelerators (linacs) are equipped with two‐ or three‐dimensional image guidance in order to correct for and minimize interfractional setup uncertainties. Image‐guided radiation therapy (IGRT) has improved accuracy for daily treatments and have allowed clinicians to decrease planning target volumes in order to spare normal tissues.^1^ However, with linac‐based treatments demanding greater accuracy, quality assurance (QA) tolerances for image‐guidance and table positioning must be in congruence with more precise treatments. The American Association in Physicists in Medicine (AAPM) task group report 142 (TG‐142) regarding quality assurance on medical accelerators specifies these tolerances with the type of treatment being delivered, especially with more complex modalities like intensity modulated radiation therapy (IMRT), stereotactic radiotherapy (SRT) and stereotactic body radiation therapy (SBRT).^2^

Conventional treatment tables are designed with four degrees‐of‐freedom (4DoF) in order to adjust positioning in the patient's vertical, lateral, and longitudinal directions, as well as an additional yaw rotation. A relatively new technology that is being outfitted on linacs are robotic six degrees‐of‐freedom (6DoF) tables that allow for mechanical adjustments of pitch and roll rotations in addition to the standard 4DoF adjustments. Early work in 6DoF corrections was investigated using BrainLAB's ExacTrac 6D system (BrainLAB AG, Feldkirchen, DE). BrainLAB utilizes a stereoscopic x‐ray system with 2D‐to‐3D registration system to reference digitally reconstructed radiographs (DRR). 6DoF registration showed a superior submillimeter localization accuracy against 3DoF registration using a head phantom.^3^ Rotational corrections with the Robotic Tilt Module on the Exactrac system showed an overall accuracy of 0.31 ± 0.77 mm with a quadrature summation of positional accuracy and isocentricity uncertainty.^4^ Takemura et al analyzed the 3D error vectors for a HexaPOD evo table (Elekta, Stockholm, SE) with and without a 60 kg weight and concluded that the additional weight did not affect the accuracy of the 6DoF positioning.^5^

Clinical indications of on‐line rotational adjustments have also been investigated. A theoretical study of rotational corrections by Ayan et al^6^ showed that depending on the shape of the target volume, the effect of not‐correcting rotational mismatches could be very detrimental dosimetrically. Both Gevaert et al^7^ and Dhabaan et al^8^ noted improved dosimetric quantities for intracranial stereotactic patients when using 6DoF corrections compared to 4DoF. 6DoF analysis of prostate treatments showed more variability. Chiesa et al. noted minimal dosimetric impact for spherical prostate targets with 6DoF corrections but potentially significant dosimetric deviations for elongated targets with seminal vesicle involvement.^9^ Other prostate studies observed rotational adjustments greater than 2° and commented on the importance to correct for larger deviations.^10^, ^11^ Two studies categorized 6DoF accuracy in terms generalized disease sites. Guckenberger et al compared 6DoF setup accuracies in terms of nonfixated immobilization (body) and fixated immobilization (cranial or head and neck).^12^ Schmidhalter et al classified 6DoF accuracies by cranial and extracranial treatments.^13^ In both studies, it was observed that extracranial, or body‐type, treatments required a larger translational and rotational correction.

While clinical implementation of 6DoF tables yield more accurate image‐guided results, routine QA for 6DoF tables has been limited. Schmidhalter et al has demonstrated reproducible 6DoF table performance using a combination of graph paper, inclinometers, and imaging methods.^14^ These tests have demonstrated a process in which routine QA for 6DoF tables can be established. While 6DoF commissioning and QA have been characterized by other studies, it is to the best of our knowledge that a streamlined procedure has yet to be developed.

One such technology that is capable of establishing an efficient workflow is 3D printing. This “relatively new, rapidly expanding” technology has advanced personalized medicine by developing customized prosthetics, models, and medical devices.^15^ Tack et al provided a systematic literature review regarding 3D printing publications in medicine.^16^ They observed a rapid rise in publications after January 2011, with a majority of the publications originating from the surgical domain in medicine. This can be exemplified by such work on 3D printed frames for laser interstitial thermotherapy,^17^ creating 3D anatomical models from magnetic resonance imaging,^18^ and accurately printing organs with heterogeneous tissues.^19^

Moreover, the emergence of 3D printing technology in various arenas in medicine has garnered similar interest in radiation oncology. A significant amount of effort into 3D printing has been utilized for patient‐specific devices, which include: bolus for electron treatments,^20^, ^21^, ^22^ compensators for photon treatments,^23^, ^24^ and patient immobilization.^25^ 3D printing in brachytherapy applicators has also been investigated. Dosimetric evaluations of an FDA‐approved material^26^ and various material infill densities^27^ has shown promising results. 3D printed phantoms have also been successfully developed for various QA demands. Ehler et al fabricated an anthropomorphic phantom to test the feasibility of rapid prototyping for patient‐specific QA.^28^ Madamesila et al characterized the variable density of 3D printed samples as a function of percent material infill that focused on densities in the lung range.^29^ From a machine‐based QA perspective, Bieniosek et al compared a 3D printed replication of a commercial PET/CT phantom to the commercial phantom itself.^30^ In terms of linac‐based QA, little effort has been explored in creating 3D printed models to meet specific QA purposes.

The aim of this work is to determine the feasibility of generating a 3D printed QA phantom in order to test the accuracy and reproducibility of a 6DoF table alignment. The 6DoF phantom will be constructed such that the tests can be performed in a streamlined fashion. The workflow will be aimed to follow typical end‐to‐end testing, where it will be CT simulated, planned for isocentric localization, setup at the treatment linac, and imaged following stereotactic IGRT protocols. After establishing baseline alignment shifts and isocentricity values, the phantom can be implemented into the daily QA routine.

## 2 MATERIALS AND METHODS

### 2.A 6DoF table

Tests were performed on three Truebeam linacs, each equipped with a 6DoF PerfectPitch table (Varian Medical Systems, Palo Alto, CA, USA). Based on relative patient coordinates, the mechanical movements possible in traditional 4DoF tables include three translational directions in the left‐right (lateral), superior‐inferior (longitudinal), and anterior‐posterior (vertical) positions, as well as a yaw rotation direction. Newer 6DoF capabilities allow for two additional rotation adjustments which can correct for the patient's pitch and roll. The PerfectPitch table is equipped with additional robotic motors to correct for pitch and roll. The pivot point for the attachment is at a different location than the yaw rotation about the machine isocenter. Thus, if only pitch and roll corrections were applied, the treatment isocenter would be rotated away from the mechanical isocenter. Restorative translational shifts need to be applied to match back to the mechanical isocenter. These restorative corrections are integrated into the image‐guided registration at the console. The mechanical limitations for pitch and roll corrections are ±3.0°.

### 2.B 3D printer

The 3D printer used was the BCN3D Sigma (BCN3D Technologies, Barcelona, ES). The Sigma model is what is commonly referred to as a relatively low‐cost “desktop” or “hobby” printer. The Sigma utilizes a fused filament fabrication (FFF) method of printing. In FFF, a spool of suitable filament material traverses a heated element and is extruded through a small diameter nozzle. During extrusion, the filament is heated past its melting point into an amorphous form and is deposited layer‐by‐layer, starting from the bottom of the model. Once deposited, the material cools and solidifies into the desired design.

The Sigma printer is a dual‐nozzle printer, allowing for a single print to be designed with two different materials or colors. The maximum build dimensions for the printer are 21.0 × 29.7 × 21.0 cm^3^. The nozzle diameter utilized was 0.4 mm. The manufacturer quoted nozzle positioning resolution was 12.5 μm in the lateral direction and 1.0 μm with the layer height. The Sigma is also equipped with a heated platform. The heated platform serves two main purposes: to mitigate warping by decreasing the cooling rate of the material and to prevent the build from detaching from the platform.

The 3D model was created using a computer‐aided design (CAD) freeware program. The final version of the design was saved as a stereolithography (STL) file. Once generated, the STL file was imported into a proprietary 3D printing preparation software and converted into a language compatible for the 3D printer called a GCODE file. A GCODE file is a numerically controlled programming language that gives multivariate instructions regarding printer extruder location, speed, temperature, and rate, along with other printer‐specific settings.

### 2.C Establishment of end‐to‐end test

**Figure 1:**
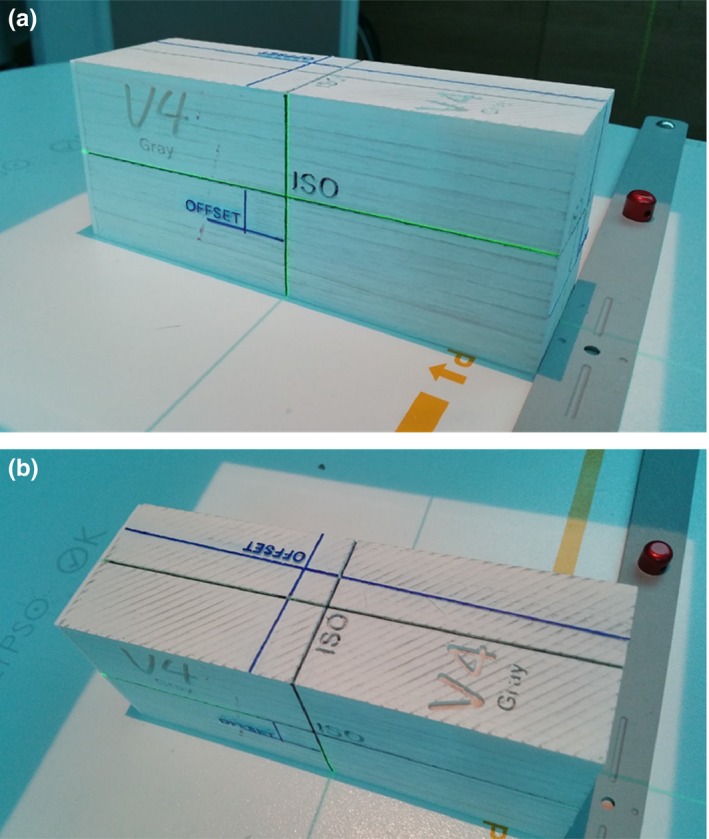
(a) Side and (b) top view of 3D printed phantom with initial setup marks (OFFSET) and isocenter indicator with BB imbedded at the center (ISO). The faces of the print are angled in order to correct for each rotation using the 6DoF couch.

In order to establish a streamlined test for 6DoF registration and mechanical motion, an end‐to‐end test was devised. Figure 1 shows one example of the 3D printed phantoms. The CAD model was built with known angular and translational offsets for alignment. The faces of the print were designed with 2.0° angular offset for the yaw, pitch, and roll rotations. An additional 3D printed leveler was also fabricated so that the skewed phantom would be leveled when used together in every rotation for reference imaging. Two sets of 1.0 mm wide lines were designed on the faces of the phantom: one for an initial offset alignment and one for final isocenter localization. With a concept similar to a hidden target test, the offset marks were used initially to set up the phantom with the lasers and field light. The offset location was designed to be exactly 1.50 cm away from the isocenter marks in each translational direction. The isocenter marks indicate the center of a 7.92 mm diameter chrome steel ball bearing (BB) that is inserted into the 3D print postfabrication. With CBCT image‐guidance, the registration software should detect the designed rotation and translation corrections from the body of the phantom. In order to facilitate the auto‐registration algorithm, unique registration structures were designed into the faces of the phantom. If registered correctly, the lasers and field light should be coincident with the isocenter lines.

Three models of the 6DoF phantom were printed and customized for the three different linacs equipped with 6DoF tables. These phantoms were to be tested on a daily basis. CT imaging for each was performed using a Discovery CT590 RT (General Electric Healthcare, Chicago, IL, USA). Each phantom was placed in the leveler for a straight alignment in order to establish a corrected reference image. The leveler was also 3D printed with a low infill percentage such that the registration algorithm would not be effected within the region‐of‐interest (ROI) of the phantom. The thinnest slice thickness (0.625 mm) was used in order to achieve the highest spatial resolution in the scan plane. The CT images were sent to Eclipse treatment planning system (Varian Medical Systems, Palo Alto, CA, USA). A plan with a CBCT setup field and six 3.0 × 3.0 cm^2^ MLC‐shaped fields was generated. The six treatment fields were used to deliver a Winston‐Lutz (WL) test^31^ at the four cardinal gantry angles and two additional collimator angles when the gantry is at 0°.

**Figure 2:**
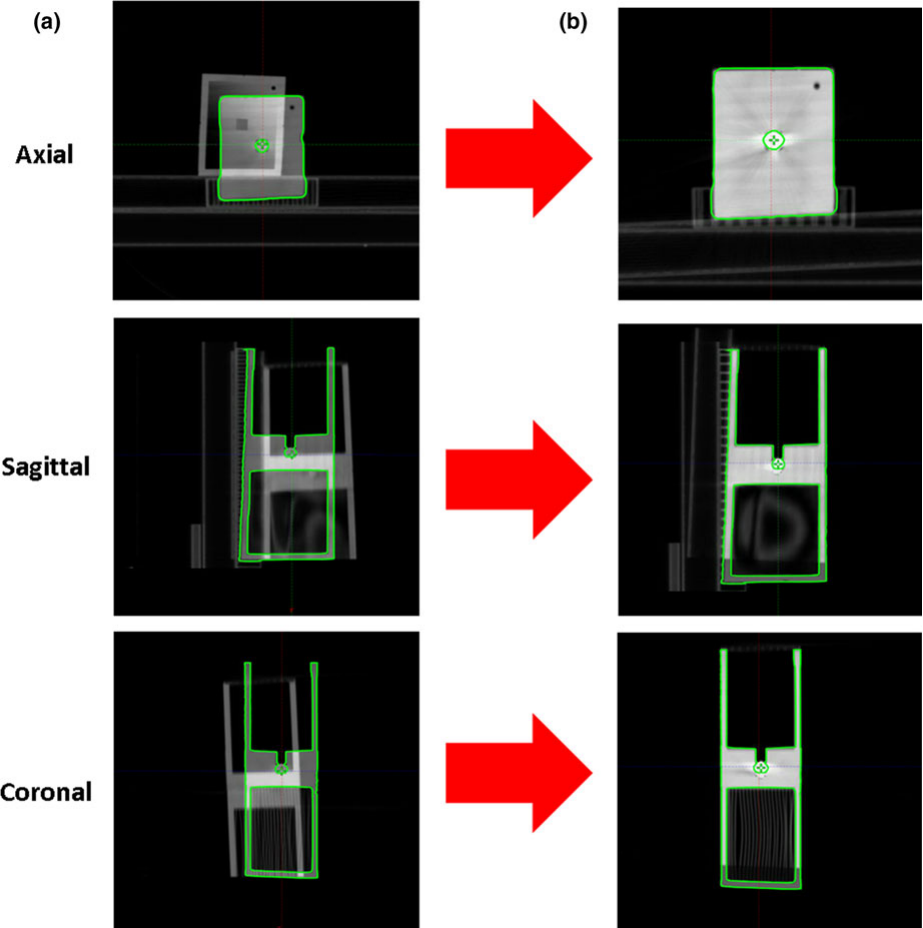
(a) An example of on‐line CBCT with initial setup of phantom in axial, sagittal, and coronal views. (b) Adjustments applied using both manual and automatic registration.

**Figure 3:**
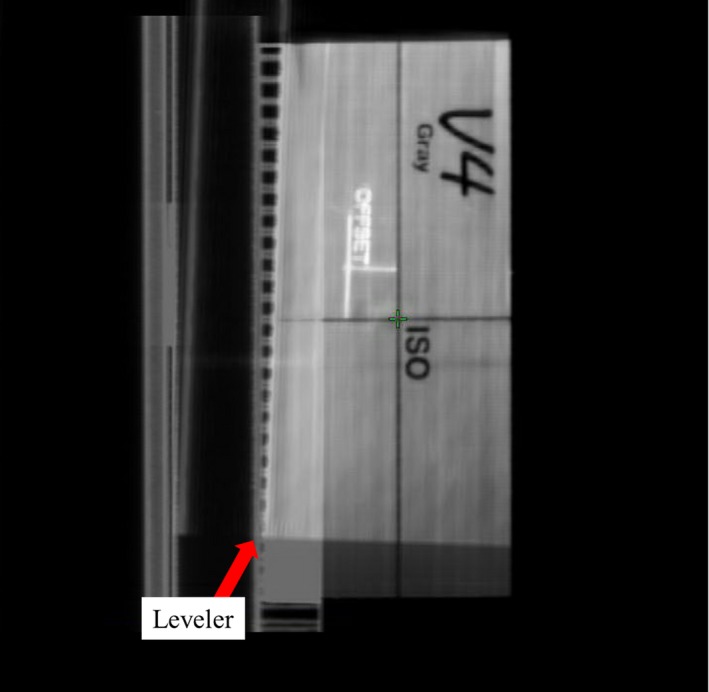
Unique registration structures shown on one face of one of the 6DoF phantom which were designed into the model. “V4 Gray” is the nickname of the linac, which has been denoted as “Linac 1” throughout the report. Note that the leveler from the reference CT is visible in the blended image.

With the leveler removed, each phantom was setup at its designated machine. Each phantom was aligned to the offset lines using the laser and field lights. A table indexer was used in order to place the phantom flush on its end for precise yaw rotation alignment. Once aligned, a half‐arc CBCT was taken. Figure 2 shows the online match of the reference CT and CBCT. A coarse, manual registration was performed only for translation adjustments. Auto‐registration was followed up for rotational and fine translational adjustments. Unique registration structures were utilized to visually verify the registration (Fig. 3). Translational and rotational shifts were then applied, with the table moving to the center of the BB at the treatment isocenter. Immediately after shifting, the six WL fields were delivered using the electronic portal imaging device (EPID). After WL delivery, laser and field light coincidence was verified with the isocenter lines indicated on the phantom. The WL test was analyzed off‐line.

The daily end‐to‐end test was carried out for 30 days for each of the three linacs. Shifts for the three translational and three rotational corrections were recorded. The displacement vectors between the center of the BB and the center of the 3 × 3 cm^2^ field for the WL tests were measured using the DoseLab Pro software (Mobius Medical Systems, Houston, TX, USA). Field and laser light verification was noted with either a pass or fail criteria given TG‐142 tolerance. Furthermore, each phantom was intercompared with each 6DoF equipped linac in order to test 3D printing reproducibility. Each individual phantom's RT DICOM reference imaging and plan was used and delivered for each linac that was tested. Five additional end‐to‐end tests were performed if the customized phantom was delivered on a different linac.

## 3 RESULTS

### 3.A 6DoF registration

**Figure 4:**
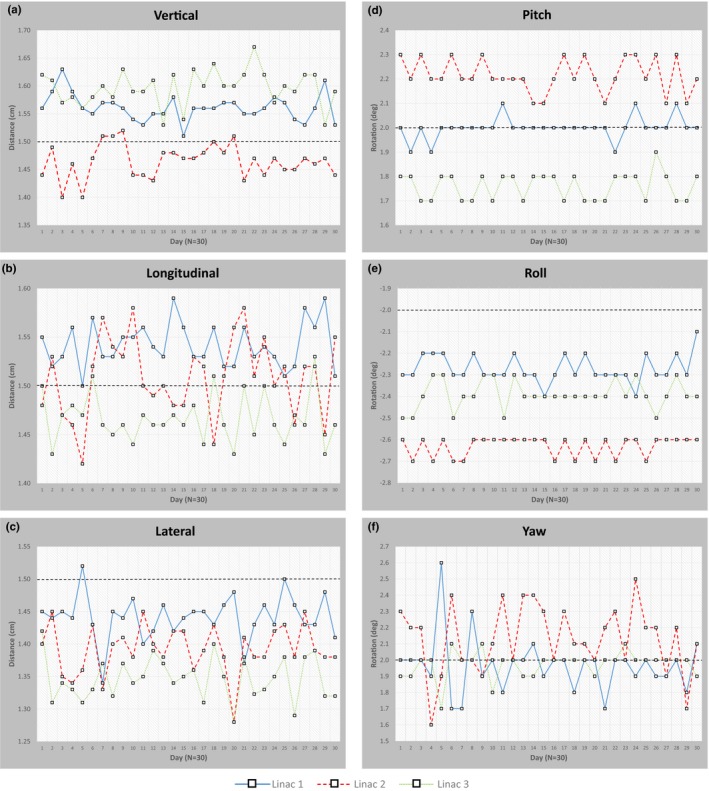
Registration values for (a) vertical, (b) longitudinal, (c) lateral, (d) pitch, (e) roll, and (f) yaw for all three linacs. Black dashed lines represents intended designed offset.

Figure 4 shows the on‐line 6DoF registration values for each linac. The dotted black line in each graph represents the designed offset values from the CAD model. Table 1 summarizes the registration data with average 6DoF values of each linac with corresponding uncertainty values of 2 standard deviations (2*σ*, or 95% confidence interval) for a 30 day data collection period (N = 30). For each individual phantom, the 2*σ* uncertainty was below 0.10 cm for each translational adjustment and 0.5° for each rotational adjustment. Combined data for all three linacs was also tabulated in Table 1 (N = 90). The combined registration values were 1.54 ± 0.13 cm, 1.51 ± 0.09 cm, 1.39 ± 0.10 cm, 2.0 ± 0.4°, −2.4 ± 0.3°, 2.0 ± 0.4° for the vertical, longitudinal, lateral, pitch, roll, and yaw adjustments respectively.

**Table 1:** Average registration values for each linac (N = 30) with 2*σ* uncertainty (95^th^ percentile)

	Vert (cm)	Long (cm)	Lat (cm)	Pitch (deg)	Roll (deg)	Yaw (deg)
Linac 1	1.56 ± 0.05	1.54 ± 0.05	1.44 ± 0.07	2.0 ± 0.1	−2.3 ± 0.1	2.0 ± 0.4
Linac 2	1.46 ± 0.06	1.51 ± 0.08	1.39 ± 0.08	2.2 ± 0.1	−2.6 ± 0.1	2.1 ± 0.4
Linac 3	1.60 ± 0.06	1.47 ± 0.05	1.35 ± 0.07	1.8 ± 0.1	−‐2.4 ± 0.1	2.0 ± 0.2
Combined (N = 90)	1.54 ± 0.13	1.51 ± 0.09	1.39 ± 0.10	2.0 ± 0.4	−2.4 ± 0.3	2.0 ± 0.4

### 3.B Winston‐Lutz test and laser/light field verification

**Figure 5:**
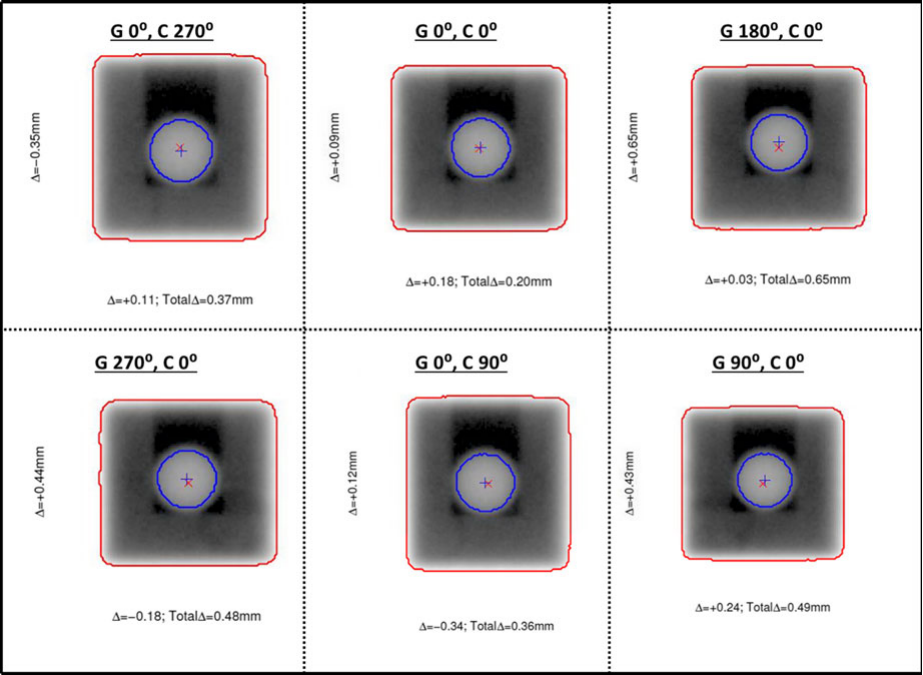
Example of Winston‐Lutz measurement with six field delivery (G=gantry angle, C=collimator angle). The blue “+” represents the center of the BB, while the red “X” represents the center of the field size.

An example of the WL analysis is shown in Fig. 5. By outlining a pixel value threshold for both the field aperture and BB, the center of each is located and both displacement vectors in the 2D coordinates can be determined at each respective WL field. The magnitude of the total displacement vector, Δ, for each field is calculated using a simple distance formula between the center of the BB and the center of the field aperture

(1) $$\Delta =\sqrt{\Delta_{x}^{2}+\Delta_{y}^{2}}$$

**Figure 6:**
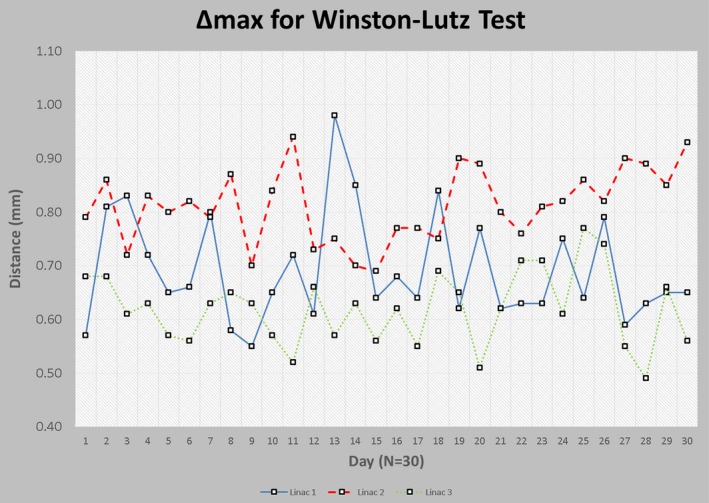
Line plot of maximum total delta (∆max) for each linac.

Δ_x_ and Δ_y_ represent the displacement vectors for the x and y direction in the planar EPID coordinate system, respectively. The field with the largest Δ would be recorded as the maximum total displacement vector, Δ_max_, which would be used as the single value for analyzing the displacement between the imaging and radiation isocenter. Δ_max_ values were recorded each day as shown in Table 2. Minimum Δ_max_, maximum Δ_max_, average Δ_max_, and uncertainty within the 95^th^ percentile (2*σ*) were tabulated. The largest Δ_max_ recorded within the 30 measurements was 0.98 mm for linac 1, and the smallest Δ_max_ recorded was 0.49 mm on linac 3. The average Δ_max_ values were 0.69, 0.81, and 0.62 mm for linacs 1, 2, and 3 respectively. The 95% confidence level was within 0.20 mm or smaller for each of the linacs. Figure 6 shows the Δmax data in a linear plot style.

**Table 2:** Maximum total delta (Δ_max_) Winston‐Lutz values for each linac (N = 30). Values in mm

	Minimum Δ_max_	Maximum Δ_max_	Average Δ_max_	2*σ*
Linac 1	0.55	0.98	0.69	0.20
Linac 2	0.69	0.94	0.81	0.14
Linac 3	0.49	0.77	0.62	0.14

After delivery of the WL test, a visual inspection of the laser and field lights were performed in order to verify that they were impinging on the 1 mm indentations of the phantom. The test was analyzed with either a pass or fail criteria. The laser and light field test passed for each linac for the 30 day period.

### 3.C Phantom intercomparison

Tables 3 and 4 show the results of the registration and WL values for intercomparison tests with each phantom to a different linac. As a reference, the values for the specific phantom to its respective linac (i.e., Phantom 1 to Linac 1) are also tabulated from the previously discussed results. The number of tests using the same phantom and linac combination (N = 30) will differ from the phantom and linac intercomparison (N = 5). Average and 2*σ* uncertainty values are tabulated for both translation and rotation adjustments.

**Table 3:** Phantom and linac intercomparison results for 6DoF registration values. Average and 2σ uncertainty (95^th^ percentile) values recorded with N = 5, except for matched phantom and linac (N = 30) displayed in grey

	Vert (cm)			Long (cm)			Lat (cm)		
	Linac 1	Linac 2	Linac 3	Linac 1	Linac 2	Linac 3	Linac 1	Linac 2	Linac 3
Phantom 1	1.56 ± 0.05	1.59 ± 0.03	1.60 ± 0.03	1.54 ± 0.05	1.54 ± 0.02	1.50 ± 0.02	**1.44** ± 0.07	1.41 ± 0.14	1.39 ± 0.05
Phantom 2	1.56 ± 0.06	1.46 ± 0.06	1.57 ± 0.04	1.46 ± 0.05	1.51 ± 0.08	1.47 ± 0.04	1.44 ± 0.07	1.39 ± 0.08	1.40 ± 0.04
Phantom 3	1.57 ± 0.05	1.61 ± 0.03	1.60 ± 0.06	1.44 ± 0.03	**1.49** ± 0.05	1.47 ± 0.05	1.40 ± 0.05	1.39 ± 0.02	1.35 ± 0.07

	Pitch (deg)			Roll (deg)			Yaw (deg)		
	Linac 1	Linac 2	Linac 3	Linac 1	Linac 2	Linac 3	Linac 1	Linac 2	Linac 3
Phantom 1	2.0 ± 0.1	2.1 ± 0.2	2.1 ± 0.2	2.0 ± 0.4	2.2 ± 0.2	1.9 ± 0.2	−2.3 ± 0.1	−2.3 ± 0.2	−2.1 ± 0.1
Phantom 2	1.9 ± 0.3	2.2 ± 0.1	2.2 ± 0.1	2.0 ± 0.0	2.1 ± 0.4	2.0 ± 0.0	−2.6 ± 0.0	−2.6 ± 0.1	−2.3 ± 0.1
Phantom 3	1.9 ± 0.3	2.2 ± 0.2	1.8 ± 0.1	1.8 ± 0.1	2.0 ± 0.0	2.0 ± 0.2	−2.6 ± 0.1	−2.6 ± 0.1	−2.4 ± 0.1

**Table 4:** Phantom and linac intercomparison results for Winston‐Lutz results. Average and 2σ uncertainty (95th percentile) values recorded with N = 5, except for matched phantom and linac (N = 30) displayed in gray

	WL (mm)		
	Linac 1	Linac 2	Linac 3
Phantom 1	0.69 ± 0.20	0.66 ± 0.16	0.49 ± 0.08
Phantom 2	0.74 ± 0.09	0.81 ± 0.14	0.67 ± 0.16
Phantom 3	0.76 ± 0.14	0.67 ± 0.17	0.62 ± 0.14

## 4 DISCUSSION

6DoF robotic tables are a relatively new technology that allows for two additional degrees of rotational freedom compared to a traditional 4DoF table. The additional degrees of freedom have the potential to deliver a more accurate treatment and could allow for a clinician to decrease target margins while sparing more normal tissue.^3^, ^4^, ^5^, ^7^, ^8^, ^9^, ^10^, ^11^, ^12^, ^13^ While there have been previous studies that have established the accuracy of various commercial 6DoF tables,^4^, ^5^, ^14^ there has yet to be a streamlined test to efficiently test the quality assurance of the robotic motion of the 6DoF table. In this study, emerging 3D printing technology was utilized in order to fabricate a phantom that could quickly and effectively test 6DoF motion in accordance with TG‐142 tolerances.

For a stereotactic linacs, TG‐142 requires table positioning/repositioning to be within 1 mm and 0.5°, mechanical and radiation isocenter coincidence within 1 mm of baseline, and laser localization to be within 1 mm.^2^ Although TG‐142 specifies these tests to be performed at different intervals, this study performed all three on a daily basis based on established institutional policy.

For the registration data that was collected over a 30 day period, each linac's phantom showed minimal uncertainty in both the translational and rotational adjustments. However, when comparing average values to the engineered offsets in the CAD model, the deviation is large enough not to use the CAD values as baseline numbers. For instance, the average values for the lateral and roll adjustments for linac 2 were 1.39 cm and 2.6° respectively. If the designed 1.50 cm and 2.0° values were used as expected values, the positioning/repositioning test would exceed the tolerance of 1.0 mm and 0.5° specified by TG‐142. However, within a 95% confidence interval, each individual linac's registration uncertainty were below the TG‐142 tolerances for both translation and rotation. Notably, the largest variations stemmed from the lateral and yaw adjustments, as there was an uncertainty ranging from 0.7–0.8 mm to 0.2–0.4° respectively. The most likely cause for this variation could manifest from the inconsistent alignment when abutting the phantom to the indexing bar. Assuming an initial 0.0° rotation in the pitch and roll, the phantom will always be flush to the table given gravitational forces. Hence, the small uncertainty observed at 0.1° for both pitch and roll. However, even if the table yaw is 0.0°, it will depend on the user to establish a flush alignment against an index bar, in which even slight yaw rotations from baseline could be corrected during the registration process. With the larger yaw uncertainty, the lateral translation adjustment would also be effected given its travel in the same plane of rotation.

When the registration statistics for each linac are combined (N = 90 for all three linacs), the uncertainty for each 6DoF adjustment increases such that either each adjustment approaches or even exceeds TG‐142 tolerances. Specifically, the 2*σ* uncertainty values increase to 1.3 mm, 0.9 mm, 1.0 mm, 0.4°, 0.3°, and 0.4° for the vertical, longitudinal, lateral, pitch, roll, and yaw adjustment respectively. Given this increase, it would be more likely that the periodic QA would fail if these combined values were used as baseline. Therefore, it is recommended that each linac's individual phantom have established baseline registration values rather than using a combined or expected value taken from a CAD model.

The modified WL test is similar to the method used by Du et al.^32^ Instead of visually aligning the phantom to the mechanical isocenter by either the laser or field lights, the isocenter was carefully defined in the treatment planning system as the center of the BB. Thus, if the online registration shifts were properly performed, the table would move the center of the embedded BB to the treatment isocenter. After delivery of the WL fields, the mechanical isocenter would be visually inspected, as it would be prior to delivery of a traditional WL test. The 1.0 mm lines designed in the phantom represented the center of the BB which would be verified with the laser and field lights.

Given this workflow, the WL test now inherently includes more uncertainties throughout the end‐to‐end process. Uncertainties originating from the reference CT and CBCT spatial positioning, random error in user dependency in localizing treatment isocenter exactly at the BB center in the treatment planning system, uncertainties arising from the registration algorithm, and any variation in table positioning are included in the overall maximum total displacement value used for the WL measurement. However, even with these additional uncertainty considerations, the results from the three linacs over a 30 day period were below 1.0 mm. The Δ_max_ range for all three linacs ranged from 0.49 to 0.98 mm, with the average falling between 0.62 and 0.81 mm. Linac 2 showed a noticeable increase in average Δ_max_ values. This is most likely machine‐specific related, as this particular stereotactic linac is 2 years older than linac 1 and 3.

Phantom and linac intercomparison tests show good agreement for both accuracy and consistency when delivering an end‐to‐end test of each customized phantom to a different linac. Using the 2*σ* uncertainty when analyzing the accuracy of individual phantoms to each linac, only the lateral adjustment for phantom 1 on linac 2 exceeded the TG‐142 specified tolerance of 1 mm table translation at 1.4 cm; all other translations were below 1 mm. The 2*σ* rotation values for all phantom and linac combinations were below the 0.5° TG‐142 tolerance. Phantom fabrication consistency is also assessed from Table 3. The range of average values for each translation and rotation adjustment are compared with each phantom on the same linac. The vertical adjustment shows the largest spread of average values, ranging from 1.46 to 1.61 cm, resulting in a 1.5 mm spread. The range of all other translation values for each linac are under 1 mm. For rotation consistency, the largest average value spread is 0.4° in pitch for linac 3. WL values were also collected for each phantom and linac combination, as presented in Table 4. All values fell below 1 mm, with the largest average value of 0.81 mm coming from phantom 2 and linac 2 combination. With this level of accuracy and consistency, the 3D printing process with a desktop printer is reproducible to be within TG‐142 tolerances. Furthermore, phantoms could be interchangeable between linacs, as long as the associated reference plan and imaging are used in conjunction with a given phantom.

Considering stereotactic delivery intent, TG‐142 specifies certain tests be performed at different periodic intervals. Laser localization should be performed daily with a 1 mm tolerance. Treatment couch position indicators should be performed monthly with a translation tolerance of 1 mm and a rotational tolerance of 0.5°. Coincidence of radiation and mechanical isocenter should be performed annually with a tolerance of 1 mm from a baseline value.^2^ Radiation and mechanical isocenter coincidence is typically tested more frequently in the form of a Winston‐Lutz test, as TG‐101 has recommended at least a monthly frequency.^33^ In this study, the tests were performed on a daily basis. By using each individual phantom's baseline data, the integrated tests for laser localization, couch position, and isocenter coincidence are within TG‐142 tolerance. Additionally, given the streamlined nature of the tests, the phantom can also be used on a daily basis with proper user training while maintaining high QA standards. It is recommended that this phantom is to be used on a daily basis while following TG‐142 tolerances for laser localization, treatment couch position, and isocenter coincidence.

It should be emphasized that the accuracy needed for the tests are dependent on the performance of the 3D printer. As of 2014, there are two dozen different varieties of 3D printing processes. Furthermore, the 3D printing market in 2014 was $700 million industry, with an increased projection of $9 billion within 10 year from that year.^34^ Thus, unlike the QA phantom market in radiation oncology, the options for 3D printers are relatively vaster. When choosing a 3D printer capable of having the same precision as a commercially available phantom, it is important to consider the specifications and additional features. Most importantly, the printer would need to provide submillimeter accuracy, as most TG‐142 tolerances for stereotactic purposes require less than or equal to 1 mm. While the quoted specifications of the printer stated sub‐millimeter accuracy, it was observed that the variance between each phantom and the designed offset dimensions were on the order of millimeter magnitude. This could stem from additional factors beyond specifications from nozzle armature precision. Amid our initial iterations of printing, the phantom would both warp and detach from the printer bed. This can be alleviated by having a printer with a heated bed and proper insulation. Commercial adhesive can also be applied to the bed of the printer in order to affix the first layer of material to the bed surface to prevent detachment. Other features like supporting structures, percent infill, and printer speed can also affect the quality of the print. While this is not an exhaustive list of considerations, the user needs to be aware of the capabilities of the 3D printer and gain experience using the printer in order to characterize its printing capacity. By fully understanding the 3D printer, one can fabricate the most optimal QA phantom.

## 5 CONCLUSION

This study investigated the use of a 3D printed phantom in order to perform a streamlined, end‐to‐end QA test on a 6DoF table. Three individual 3D printed phantoms for three linacs were fabricated with known translational and rotational offsets from a central BB. The phantom was CT simulated in a corrected orientation as a reference. A plan was created with a CBCT setup field followed by WL fields for mechanical and radiation isocenter verification using an EPID. The phantom was setup to the designed offset marks, cone‐beamed and 6DoF registered, delivered and analyzed the WL fields, and verified proper positioning/repositioning with alignment marks indicating isocenter for 30 days at each linac. Registration uncertainty values were below TG‐142 translation and rotation tolerances for each linac. Maximum total displacement values for WL analysis were below 1.0 mm, and laser and light field verification passed for each linac. With an acceptable 3D printer with submillimeter accuracy, a QA phantom can be constructed that can efficiently test the robotics of a 6DoF table.

## CONFLICT OF INTEREST

The authors disclose no conflict of interest.
